# Pulmonary infection by non-tuberculous mycobacteria in an endemic region for tuberculosis in Northeast Brazil

**DOI:** 10.1590/1414-431X2026e15340

**Published:** 2026-07-03

**Authors:** A.S. Lima, W.H.V. Carvalho-Silva, K.M. Gomes, R.S. Duarte, C.F. Luna, H.C. Schindler, L.M.L. Montenegro

**Affiliations:** 1 Departamento de Imunologia, Instituto Aggeu Magalhães, Fundação Oswaldo Cruz, Recife, PE, Brasil; 2 Secretaria de Saúde e Vigilância Ambiental, Ministério da Saúde, Brasília, DF, Brasil; 3 Departamento de Microbiologia Médica, Instituto de Microbiologia Paulo de Góes, Universidade Federal do Rio de Janeiro, Rio de Janeiro, RJ, Brasil; 4 Núcleo de Estatística e Geoprocessamento, Instituto Aggeu Magalhães, Fundação Oswaldo Cruz, Recife, PE, Brasil; 5 Centro Universitário Facol, UNIFACOL, Vitória de Santo Antão, PE, Brasil

**Keywords:** Pulmonary non-tuberculous mycobacteriosis, Pulmonary diseases, Tuberculosis, Differential diagnosis, Antimicrobial resistance

## Abstract

A wide range of non-tuberculous mycobacteria (NTM) species have been identified worldwide, with increasing recognition of their clinical importance in pulmonary disease. To evaluate the diversity, frequency, antimicrobial resistance profile, and clinical characteristics of pulmonary NTM cases in Pernambuco, Brazil, we conducted a descriptive clinical-epidemiological and comparative cross-sectional study of pulmonary NTM (PNTM) along with pulmonary tuberculosis (PTB). Pulmonary biological samples were obtained and analyzed by bacilloscopy, culture, and biochemical tests for PNTM and PTB diagnosis. Molecular analysis for *hsp65* and *rpoB* genes was also performed to confirm NTM species and classified as slowly growing mycobacteria (SGM) or rapidly growing mycobacteria (RGM). Antimicrobial susceptibility tests were also performed with 13 different drugs. Twenty-one PNTM cases were identified. They were significantly associated with a previous history of TB (66.7%) and occurred more frequently in males (67.7%). The age of the PNTM group (52.0±14.7 years) was significantly higher than PTB group (44.0±14.8 years; P=0.006). Six different NTM species were detected (three SGM and three RGM): *M. kansasii* (57.1%), *M. intracellulare* (9.5%), *M. abscessus* subsp. *abscessus* (9.5%), *M. abscessus* subsp. *bolletii* (9.5%), *M. fortuitum* (9.5%), and *M. asiaticum* (4.8%). Among the cases, 15 specimens demonstrated antimicrobial resistance to at least one drug. This is the first study to describe the frequency, diversity, and antimicrobial resistance of NTM species associated with pulmonary disease in Pernambuco, providing relevant epidemiological data to support diagnosis and management in this endemic region for tuberculosis.

## Introduction

Currently, the genus *Mycobacterium* is composed of various species, including the nine that constitute the *Mycobacterium tuberculosis* complex (MTBC), all capable of causing tuberculosis (TB) in humans. In addition to *M. tuberculosis* itself, the MTBC includes *M. bovis*, *M. bovis-BCG*, *M. africanum*, *M. microti*, *M. caprae*, *M. pinnipetti*, *M. canetti*, and *M. orygis* (1,2). The genus also encompasses *M. leprae* and a diverse group of species collectively known as non-tuberculous mycobacteria (NTM), or atypical mycobacteria (2). NTMs exhibit distinct phenotypic, genetic, and pathogenic characteristics and are further classified into rapidly growing mycobacteria (RGM) and slowly growing mycobacteria (SGM) as proposed by Runyon (1959, doi: 10.1016/s0025-7125(16)34193-1) (3,4).

NTMs are ubiquitous in the environment, having been isolated from water (including treated supplies), soil, animals, surgical equipment, and disinfectant solutions. Human infection usually occurs through inhalation, inoculation, or ingestion of contaminated material, potentially leading to pulmonary disease or surgical site infections, although person-to-person transmission does not appear to occur (3,5,6). Clinically, NTM disease may present as pulmonary, lymphatic, cutaneous, or disseminated forms, with pulmonary involvement being the most common, particularly among individuals with preexisting lung conditions such as pneumoconiosis, chronic obstructive pulmonary disease (COPD), prior tuberculosis, chronic bronchitis, bronchiectasis, or esophageal disorders associated with chronic aspiration (5,7).

The geographic distribution of NTM species associated with diseases varies considerably across different global regions. *M. kansasii* is frequently found in pulmonary infections in the United States, European countries, and South Africa. In addition, species belonging to the *Mycobacterium avium* complex (MAC), including *M. avium*, *M. intracellulare*, *M. colombiense*, and *M. chimaera*, have significant clinical relevance worldwide (3,4,6,8). In various regions of Brazil, a wide diversity of NTM species has also been isolated from pulmonary cases. The most frequently identified species include *M avium*, *M. intracellulare*, *M. kansasii*, *M. fortuitum*, *M. chelonae*, *M*. *abscessus*, and *M. massiliense* (9-11).

The clinical evaluation of pulmonary disease caused by NTM is often challenging due to the similarity of its symptoms with those of preexisting pulmonary diseases. The signs and symptoms associated with NTM-related pulmonary diseases are variable and nonspecific, and in most cases, the clinical presentation resembles the chronic progression of tuberculosis (12,13).

The differential diagnosis between TB and other mycobacterioses is crucial in TB-endemic regions, as these diseases exhibit distinct epidemiological patterns, prognoses, and treatment approaches. Therefore, the involvement of a specialized laboratory with appropriate infrastructure for species isolation and identification is essential (2,4,14,15). Caution is required during the diagnosis process, as the isolation of NTM from non-sterile clinical specimens may reflect transient colonization or contamination rather than true infection (15). The Brazilian Guidelines and the American Thoracic Society recommend that the diagnosis of mycobacteriosis be based on a combination of bacteriological, clinical, and radiological criteria (2,16).

Infections caused by NTM are increasing worldwide; however, the magnitude and regional distribution of these cases in TB-endemic countries remain poorly understood (3,6). In Brazil, most reported cases of NTM infections are concentrated in the southeastern region (11,17,18). Studies conducted in the northern region showed that a wide variety of NTM species may be associated with pulmonary disease and highlighted the importance of differentiating TB from NTM to ensure appropriate treatment (9,10). In contrast, in the northeastern region, there is a lack of studies providing epidemiological data or information regarding the diversity of NTM species involved in pulmonary disease.

Therefore, this study aims to investigate the clinical and epidemiological profiles of pulmonary cases caused by NTM compared to pulmonary tuberculosis. In addition, it seeks to evaluate the diversity, frequency, and antimicrobial resistance profile of NTM species isolated from residents of a TB-endemic state in Northeast Brazil.

## Material and Methods

### Study design

We conducted a descriptive clinical-epidemiological study of pulmonary non-tuberculous mycobacteriosis (PNTM) cases, along with comparative cross-sectional analyses involving individuals diagnosed with pulmonary tuberculosis (PTB).

All patients received detailed information about the research and were invited to participate voluntarily. They completed standard questionnaires, gave written informed consent, and provided biological samples for laboratory analysis. This study was approved by the Research Ethics Committee of the Aggeu Magalhães Institute - IAM/FIOCRUZ (CAAE: 07382012.4.0000.5190; approval protocol number: 220.364).

### Selection of PNTM cases and clinical specimens

The inclusion criteria were patients aged 18 years or older, of both sexes, with suspected mycobacterial pulmonary disease regardless of bacilloscopy results. NTM were isolated from at least two pulmonary samples (sputum and/or bronchoalveolar lavage). Species identification was performed using phenotypic, biochemical, and molecular techniques. Clinical diagnosis was confirmed by a health service physician in a blinded manner, following guidelines (15,16). Confirmed PNTM cases were referred to the bacteriology sector of the Central Public Health Laboratory of Pernambuco (LACEN - PE), where cultures and differentiation between MTBC and NTM were performed. Clinical, epidemiological, and laboratory data were obtained from the State Health Department's electronic database (Ambulatory and Laboratory Manager - ALM) and from patients' medical records. The exclusion criteria were prior initiation (before sample collection) of NTM or TB treatment; insufficient biological samples for laboratory processing; samples showing culture contamination; isolation of NTM in only one of multiple respiratory samples with negative results in the remaining cultures; and absence of essential clinical information, treatment data, or follow-up records in the ALM system or in patients' medical records, which precluded definitive diagnostic confirmation.

### Selection of pulmonary tuberculosis cases

Fifty-five individuals aged 18 years or older, of both sexes, with clinical and/or radiological evidence of PTB and confirmed by isolation of *M. tuberculosis* from sputum and/or bronchoalveolar lavage, were included. Diagnosis was established via direct bacilloscopy and/or culture, or by evident clinical improvement after specific treatment, as recommended by the Brazilian guidelines (15). The exclusion criterion was insufficient clinical or laboratory data to establish the final diagnosis.

### Bacilloscopy, culture, and biochemical tests

Bacilloscopy was performed using the Ziehl-Neelsen method to identify and quantify acid-alcohol resistant bacilli (AARB). Clinical specimens were decontaminated by Petroff's method (4% NaOH), inoculated in Lowenstein-Jansen medium, and incubated at 37°C in the dark for up to eight weeks. Differentiation between MTBC and NTM was based on colony morphology, cord factor production, susceptibility to para-nitrobenzoic acid (PNB), niacin production, and catalase activity at 68°C. All procedures were conducted in accordance with Brazilian guidelines for laboratory diagnosis (15).

### Molecular identification

All isolates were also identified by specific gene sequencing (*hsp65* and *rpoB*) for confirmation and classification of SGM and RGM. The methodology followed a previously published study (19). For *hsp65* gene, the primers (F: 5-GGCCAAGACAATTGCGTACG-3 and R: 5-GGAGCTGACCAGCAGGATG-3) were used to amplify a sequence of 667 bp. The final PCR volume reaction was 25 µL and contained: DNA solution (50 ng); 10x PCR buffer, 1.5 mM MgCl_2_, 2 mM dNTP, 10 µM of each primer, 1.25 U of Platinum Taq DNA polymerase (Invitrogen, USA), and ultrapure water to complete the volume. Amplification conditions were 2 min at 95°C, followed by 30 cycles of 94°C for 45 s, 57°C for 45 s, and 72°C for 45 s, with a final extension step at 72°C for 5 min. For the *rpoB* gene, a 764-bp fragment was amplified and sequenced with primers: F: 5-GCAAGGTCACCCCGAAGGG-3 and R: 5-AGCGGCTGCTGGGTGATCATC-3. The final PCR reaction volume was also 25 µL, containing: DNA solution (50 ng), 10x PCR buffer, 2.5 mM MgCl_2_, 2 mM dNTP, 10 µM of each primer, 1.0 U of Platinum Taq DNA polymerase (Invitrogen), and ultrapure water to complete the volume. PCR mixtures were heated at 95°C for 1 min and then subjected to 35 cycles of denaturation at 94°C for 30 s, annealing at 64°C for 30 s, and extension at 72°C for 90 s, with a final step at 72°C for 5 min. PCR products were loaded onto 2% agarose gel for electrophoresis. Amplicons were then purified with GFX PCR DNA and Gel Band Purification Kit (G&E, USA) and sequenced on an ABI PRISM 3100 sequencer with a BigDye Terminator cycle sequencing kit (Applied Biosystems, USA). The sequences obtained were compared with those deposited in the GenBank NCBI database by using the BLAST software.

### Antimicrobial susceptibility testing

Susceptibility testing was performed by broth microdilution according to CLSI guidelines (20). Broth microdilution is a highly accurate antimicrobial susceptibility testing (AST) method that determines the minimum inhibitory concentration (MIC) by exposing microorganisms to serial dilutions of antibiotics in liquid culture media, with the MIC being the lowest concentration that inhibits visible growth. Testing must be performed using recent primary cultures (≤4-5 weeks) with sufficient growth to allow proper inoculum standardization. SGM species were tested against amikacin, ciprofloxacin, clarithromycin, moxifloxacin, sulfamethoxazole, rifampicin, ethambutol, isoniazid, and streptomycin. RGM species were tested against amikacin, cefoxitin, ciprofloxacin, clarithromycin, doxycycline, moxifloxacin, sulfamethoxazole, and tobramycin.

### Statistical analysis

Descriptive statistics were used to characterize the groups in terms of sociodemographic, clinical, and laboratorial profiles. Bivariate analysis was performed using Fisher's exact test or chi-squared test (for categorical variables) and Student's *t*-test (for normally distributed numerical variables) after the normality test by Shapiro-Wilk. All tests were two-tailed, and the significance level (α) of 0.05 was adopted. Analyses were conducted using SPSS software (version 30, IBM, USA) and R program (version 4.4.2).

## Results

### Patient characteristics

Over the three years of the study, LACEN - PE received 4,808 pulmonary samples from patients with suspected mycobacterial infections. Among the positive samples (32.8%), 1,506 (95.4%) were identified as MTBC, while 72 (4.6%) yielded NTM, corresponding to 33 individuals. Of these, 21 (63.6%) met the criteria for PNTM, as they presented at least two positive sputum cultures or one positive bronchoalveolar lavage with isolation of the same NTM species, in addition to clinical and respiratory manifestations consistent with mycobacterial infection (15). The remaining 12 individuals (36.4%) could not be confirmed as PTNM cases due to one or more of the following limitations: a) only one pulmonary sample was submitted for laboratory testing; b) NTM was isolated in only one of multiple samples, while the others were negative; or c) clinical data, treatment information, or follow-up were unavailable in the ALM system or in patients' medical records at the time of data collection.

Among the 21 confirmed cases of pulmonary mycobacteriosis, only seven (33.3%) received antimicrobial treatment specifically targeting NTM. The remaining 14 (66.7%) were misdiagnosed with TB and treated with the standard anti-TB regimen prescribed by health service physicians. The epidemiological and laboratory characteristics of the 21 patients with PNTM are summarized in Table 1.

**Table 1:** Characteristics of the pulmonary non-tuberculous mycobacteria cases enrolled in this study.

No.	Sex	Age	Occupation	Area	AFB smear (+,-)	Positive cultures (n)	Clinical sample	Other associated conditions	Species	Growth category
1	M	56	retiree	urban	+	3	sputum	cancer	*M. kansasii*	SGM
2	F	34	case-worker	urban	+	3	sputum	-	*M. kansasii*	SGM
3	F	49	dentist	urban	+	2	sputum	-	*M. abscessus subsp bolletii*	RGM
4	F	61	retiree	urban	+	3	sputum	previous tuberculosis	*M. fortuitum*	RGM
5	M	75	retiree	urban	-	3	sputum	previous tuberculosis	*M. abscessus subsp abscessus*	RGM
6	M	54	driver	urban	+	1	BAL	previous tuberculosis	*M. kansasii*	SGM
7	F	44	housewife	urban	+	2	sputum	previous tuberculosis	*M. asiaticum*	SGM
8	M	32	unemployed	urban	+	4	sputum	previous tuberculosis	*M. kansasii*	SGM
9	M	28	general services	urban	-	8	sputum	previous tuberculosis	*M. kansasii*	SGM
10	M	55	retiree	urban	-	2	sputum	previous tuberculosis	*M. kansasii*	SGM
11	M	63	merchant	urban	-	2	sputum	-	*M. kansasii*	SGM
12	F	43	retiree	urban	+	4	sputum	-	*M. kansasii*	SGM
13	M	34	unemployed	urban	-	2	sputum	previous tuberculosis	*M. kansasii*	SGM
14	M	59	retiree	urban	+	2	sputum	-	*M. abscessus subsp abscessus*	RGM
15	F	58	retiree	urban	+	2	sputum	previous tuberculosis	*M. kansasii*	SGM
16	M	47	building inspector	urban	+	2	sputum	previous tuberculosis	*M. kansasii*	SGM
17	M	69	retiree	urban	+	3	sputum	COPD and previous tuberculosis	*M. intracellulare*	SGM
18	M	57	attendant	urban	-	3	sputum	-	*M. fortuitum*	RGM
19	M	69	driver	urban	+	3	sputum	previous tuberculosis	*M. kansasii*	SGM
20	F	74	retiree	urban	+	3	sputum	previous tuberculosis	*M. abscessus subsp bolletii*	RGM
21	M	28	merchant	urban	+	2	sputum	previous tuberculosis	*M. intracellulare*	SGM

AFB: acid fast bacilli; BAL: bronchoalveolar lavage; COPD: chronic obstructive pulmonary disease; RGM: rapidly growing mycobacteria; SGM: slowly growing mycobacteria.

The PNTM group had a significantly higher mean age than the PTB group (52.0±14.7 *vs* 41.0±14.8 years; P=0.006). More than half of the PNTM patients were aged ≥57 years (12/21; 57%), and 67.7% (14/21) were male compared to 70.9% in the PTB group. Diagnostic delay was frequent in PNTM: 11 patients (52.4%) were diagnosed after six months and 9 (42.9%) after ≥1 year of symptoms. Smoking (57 *vs* 23.5%; P=0.012) and alcohol use (38 *vs* 18.2%; P=0.127) were more common in PNTM than in PTB. Comorbidities were also observed in the PNTM and PTB groups. History of TB was observed in 66.7% of PNTM patients *versus* 10.1% in the PTB group (P<0.001). Interestingly, none of the PNTM patients were people living with HIV (PLHIV), while 30.9% of PTB patients were also diagnosed with HIV (P=0.010). Cancer and COPD were each observed in one PNTM patient. Regarding clinical manifestations, cough and weight loss were the most frequent symptoms in both groups. In PNTM, productive cough occurred in 66.7% (14/21) and hemoptysis reported in one case (4.8%), compared to 61.8 and 7.3%, respectively, in the PTB group. Weight loss was reported in 66.7% of PNTM patients and in 83.6% of PTB patients, while fever was observed in 42.8 and 72.7%, respectively. Most PNTM patients (92.5%) had no inflammatory signs such as pain, redness, or swelling (Table 2). Sociodemographic and clinical characteristics of the PNTM and PTB groups are also provided in Supplementary Table S1.

**Table 2:** Clinical symptoms and X-ray alterations of nine patients with pulmonary non-tuberculous mycobacteria.

No.	Clinical symptoms	Chest X-ray
2	Dry cough, weight loss, no fever or signs of inflammation	Pleural thickening and infiltrate
3	Productive cough, weight loss, no fever or signs of inflammation	Bronchiectasis
8	Productive cough, weight loss, fever, no signs of inflammation	Cavity
11	Productive cough, weight loss, fever, no signs of inflammation	Bronchiectasis, fibrosis, and pulmonary nodules
13	Dry cough, weight loss, fever, no signs of inflammation	Bronchiectasis
14	Productive cough, weight loss, fever, no signs of inflammation	Pleural thickening fibrosis
17	Productive cough, weight loss, no fever or signs of inflammation	Bronchiectasis, fibrosis, cavity, and infiltrate
20	Productive cough, weight loss, fever, no signs of inflammation	Bronchiectasis
21	Productive cough, weight loss, fever, and presence of lymph nodes in the neck	Cavity

Radiological (chest X-ray) alterations were observed in all PNTM patients; however, detailed records were available for only 9 patients (42.9%). Among these, bronchiectasis (55.6%) was most frequent, followed by fibrosis (33.3%), cavitation (33.3%), pulmonary infiltrate (22.2%), pleural thickening (22.2%), and pulmonary nodules (11.1%). Nearly half of these patients (44.4%) exhibited more than two types of radiological alterations (Table 2). For the other 12 PNTM patients, there was no detailed information in medical records regarding radiological alterations, and in these cases, clinical evaluation was not performed.

### NTM isolates

Six different NTM species (three RGM and three SGM) were identified from cultures of PNTM clinical samples according to guidelines (15,16). The most frequent species was *M. kansasii*, detected in 12 cases (57.1%), followed by *M. intracellulare* (2; 9.5%), *M. abscessus* subsp. *abscessus* (2; 9.5%), *M. abscessus* subsp. *bolletii* (2; 9.5%), *M. fortuitum* (2; 9.5%), and *M. asiaticum* (1; 4.8%). Pulmonary infection by *M. asiaticum* and *M. abscessus* subsp. *bolleti* occurred exclusively in women, with a mean age of 55.7 years. Among men, *M. kansasii* predominated, identified in 10 cases (83.3%) with a mean age of 48.1 years, and *M. intracellulare* occurred also exclusively in men with a mean age of 48.5 years. Among the 12 individuals who did not meet the criteria for PNTM (15,16), the isolated species included *M. fortuitum*, *M. kansasii*, *M. abscessus* subsp. *bolleti*, *M. abscessus* subsp. *abscessus*, and *M. szulgai*.

### Susceptibility tests for antibiotics

Antimicrobial susceptibility testing was performed on 15 of 21 (71.4%) NTM isolates to assess their sensitivity and resistance to drugs commonly used in clinical practice for SGM and RGM. Six isolates were excluded because the cultures were older than five weeks or did not show adequate growth in liquid medium after subculturing, which precluded reliable assay performance. Among the SGM, 10 isolates of *M. kansasii* and one of *M. intracellulare* were tested. In the RGM group, two isolates of *M. fortuitum*, one of *M. abscessus* subsp. *bolletii* and one of *M. abscessus* subsp. *abscessus* were analyzed. Notably, all tested isolates - both SGM and RGM - demonstrated *in vitro* susceptibility to amikacin. Supplementary Table S2 summarizes the antimicrobial resistance profiles of these clinical isolates.

## Discussion

In this study, 21 individuals were characterized as cases of pulmonary NTM based on AST, sequencing, and the criteria established by the guidelines (15,16), representing 63.6% of all NTM-positive cultures. The remaining 12 cases (36.4%) did not meet the diagnostic criteria for pulmonary NTM infection. However, this does not exclude the possibility of disease, as these patients were not followed up, preventing definitive confirmation. As highlighted by previous authors (12,16), close monitoring is essential, since limited knowledge of NTM pathophysiology still hinders the distinction between colonization and slowly progressing infection.

Regarding the risk factors associated with NTM-related diseases, male sex, advanced age, smoking, and a previous history of TB were the most frequently observed factors in this study. Several specific risk factors for pulmonary NTM disease have been consistently reported, including chronic pulmonary disease, advanced age, sex, HIV infection, and prior TB, with the latter historically regarded as one of the most important (6,12). Recent systematic reviews and meta-analyses have confirmed that advanced age, male sex, and history of TB are strongly associated with both disease progression and increased mortality in NTM pulmonary disease (13,21,22). These findings suggest that a history of TB may facilitate NTM infection due to pulmonary alterations caused by *M. tuberculosis* and malnutrition, which is often associated with this disease (23,24). In addition, cancer and COPD were less frequent in this cohort (one case each), consistent with findings in the north of Brazil (9).

In our cohort, males were more frequently affected in both groups (PNTM and PTB), which aligned with studies suggesting a higher prevalence in men (6,25), but contrasted with other reports describing female predominance, particularly in nodular/bronchiectatic disease caused by SGM (13). Interestingly, species-specific analysis revealed that *M. abscessus* subsp. *bolletii* caused disease exclusively in women. This observation is consistent with the findings of Griffith et al. (26), who reported a predominance of females among cases of pulmonary disease caused by RGM. These studies suggest that sex-related biological and behavioral factors may influence species distribution.

The mean age of pulmonary NTM cases in this study was 52 years, with 57% over 57 years old, which is in agreement with previous findings in Brazil and worldwide (6,10,27) and supported by more recent epidemiological data reporting that older age remains one of the strongest predictors for NTM pulmonary disease (3,6).

Radiological patterns in pulmonary NTM disease are commonly classified as cavitary (“classical”) or nodular with bronchiectasis (“non-classical”). However, recent imaging reviews show a broader spectrum, with a variety of manifestations beyond these two forms (28). A Brazilian review also highlights that cavitation (88.9%), bronchiectasis (77.8%), and pulmonary nodules (55.6%) are frequently observed, often localized to the middle lobe and lingula, features that may better reflect the complexity of NTM presentations (29). In our cohort, 44% of patients showed more than two distinct radiographic alterations, placing them outside traditional classifications, and also corroborated by another study in Brazil (10).

In our study, most patients with pulmonary NTM were initially treated with the standard TB regimen, and the correct diagnosis of NTM disease was often delayed, frequently occurring several months after symptom onset. These findings highlight the importance of differential diagnosis between PTB and PNTM, including accurate species identification. In TB-endemic countries, however, delays in diagnosing and identifying NTM species remain common, largely due to limited laboratory infrastructure and the prioritization of TB-focused programs. As a result, many patients are managed solely based on clinical presentation and smear microscopy, often receiving inappropriate treatment that may contribute to antimicrobial resistance (30). More recent studies confirm that misdiagnosis of NTM as TB remains a critical challenge in high-burden settings, where insufficient diagnostic capacity and reliance on clinical features frequently lead to delayed or inadequate therapy (6,31). Such delays not only hinder the initiation of appropriate therapy but may also worsen patient outcomes and increase the risk of treatment failure.

Because NTM diseases are not transmissible, they are generally not subject to mandatory notification, except in cases of healthcare-associated outbreaks, such as post-surgical infections. This results in a lack of official records capable of accurately estimating their prevalence in the country (12,32). Likewise, there is a shortage of epidemiological data on the diversity and frequency of NTM species associated with pulmonary disease, particularly in TB-endemic countries, where regional studies remain insufficient to assess the true burden of NTM infections (30,31).

The identification of NTM species associated with pulmonary disease is essential for guiding diagnosis, therapy, and health service organization (9,11). In this study, phenotyping of isolates from culture and the sequencing of *hsp65* and *rpoB* genes revealed *M. kansasii*, *M. intracellulare*, *M. abscessus* subsp. *abscessus*, *M. abscessus* subsp. *bolletii*, *M. fortuitum*, and *M. asiaticum* as the most frequent isolates.

The prevalence of NTM species varies considerably across geographical regions worldwide. In most global regions, the *M. avium* complex (which includes *M. intracellulare*) is the most frequently isolated NTM, followed by the *M. abscessus* complex, *M. fortuitum*, and *M. simiae*, a pattern consistently reported in the literature (6,8,30,33). In South America, however, the *M. avium* complex and *M. kansasii* are more commonly identified (3,6), and many regions in Brazil follow this epidemiological pattern. In our study, the distribution of these species was similar to reports from Southeastern Brazil and Bahia state (Northeast) but differed from findings in Pará state (North), where *M. massiliense* and *M. simiae* were more prevalent, likely reflecting environmental influences (9). Notably, we identified a case of PNTM caused by *M. asiaticum*, a rarely reported species, with only isolated cases worldwide and no standardized treatment guidelines. Its antimicrobial susceptibility pattern is not well established due to the limited number of reported infection cases. This NTM was first described as a cause of disease in humans in 1980, and since then, only a few cases have been described in the literature (34,35). The isolate identified as *M. asiaticum* in this study was obtained from a female patient with a clinical presentation suggestive of PTB, characterized by a productive cough lasting more than 15 days and fever. She was treated with the standard tuberculosis regimen for six months and showed a favorable therapeutic response.

Species identification also plays a central role in therapy, since it provides the first indication of antimicrobial susceptibility (36). However, *in vitro* susceptibility does not always predict clinical response, especially for *M. avium*, *M. abscessus*, and other slow-growing NTM (36,37). In our study, *M. abscessus* subsp. *abscessus* and *M. abscessus* subsp. *bolletii* showed resistance to most drugs, with activity observed only for amikacin against both species. These findings are consistent with other reports highlighting the intrinsic resistance and poor treatment outcomes of *M. abscessus*, often requiring prolonged multidrug regimens and sometimes surgical resection (16,38).

Other species showed more favorable therapeutic responses. *M. fortuitum*, although more commonly associated with skin and soft tissue infections, has been reported in pulmonary disease and generally responds to at least two active agents for 12 months or longer (39,40). In our cohort, patients with *M. fortuitum* responded well to prolonged multidrug therapy. *M. kansasii* usually presents good clinical outcomes, with susceptibility to rifampicin, macrolides, and fluoroquinolones. In our study, only one isolate was resistant to rifampicin, but clinical improvement was achieved with a clarithromycin-based regimen. These findings align with current evidence indicating that treatment success is higher for *M. kansasii* than for other NTM species (16).

In conclusion, this study highlighted the need to improve epidemiological surveillance strategies for infections caused by NTM, particularly in TB-endemic countries. We reported for the first time in the state of Pernambuco the frequency and diversity of NTM species associated with pulmonary diseases, as well as assessed the true magnitude of this pathology in a region known to be endemic for TB. Our findings demonstrated that several NTM species are involved in pulmonary infections in Pernambuco. In this context, it is essential to establish a differential diagnosis between PTB and PNTM disease, particularly in individuals with a history of TB or treatment failure. As NTM diseases are not subject to compulsory notification, the incorporation of diagnostic confirmation and differential diagnosis into national surveillance systems is essential to avoid misdiagnosis and inappropriate treatment, often based on TB regimens, which may lead to therapeutic failure, unnecessary antimicrobial exposure, adverse events, and the selection of antimicrobial resistance. Furthermore, expanding access to molecular identification methods is critical for accurate species-level diagnosis, timely initiation of appropriate therapy, improved clinical outcomes, and optimization of public health resources. Finally, the integration of clinical data from healthcare services with laboratory results from reference centers would strengthen epidemiological monitoring and support more effective clinical decision-making and public health policies.

## Data Availability

All data generated or analyzed during this study are included in this published article.

## Supplementary Materials

Supplementary Material Click to view [pdf].

## References

[1] Gagneux S (2018). Ecology and evolution of *Mycobacterium tuberculosis*. Nat Rev Microbiol.

[2] Ministério da Saúde (2022). Manual nacional de vigilância laboratorial da tuberculose e outras micobactérias.

[3] Cowman S, van Ingen J, Griffith DE, Loebinger MR (2019). Non-tuberculous mycobacterial pulmonary disease. Eur Respir J.

[4] Xu N, Li L, Wu S (2024). Epidemiology and laboratory detection of non-tuberculous mycobacteria. Heliyon.

[5] Nqwata L, Ouédrago AR (2022). Non-tuberculous mycobacteria pulmonary disease: a review of trends, risk factors, diagnosis and management. Afr J Thorac Crit Care Med.

[6] Prevots DR, Marshall JE, Wagner D, Morimoto K (2023). Global epidemiology of nontuberculous mycobacterial pulmonary disease: a review. Clin Chest Med.

[7] Chin KL, Sarmiento ME, Alvarez-Cabrera N, Norazmi MN, Acosta A (2020). Pulmonary non-tuberculous mycobacterial infections: current state and future management. Eur J Clin Microbiol Infect Dis.

[8] Dahl VN, Mølhave M, Fløe A, van Ingen J, Schön T, Lillebaek T (2022). Global trends of pulmonary infections with nontuberculous mycobacteria: a systematic review. Int J Infect Dis.

[9] da Costa ARF, Luiza M, De Sousa MS, Noel P, Sales LHM, Lim KVB, Amal A (2012). Pulmonary Infection, InTech.

[B10] da Costa ARF, Falkinham JO, Lopes ML, Barretto AR, Felicio JS, Sales LHM (2013). Occurrence of nontuberculous mycobacterial pulmonary infection in an endemic area of tuberculosis. PLoS Negl Trop Dis.

[11] Marques LRM, Ferrazoli L, Chimara É (2019). Pulmonary nontuberculous mycobacterial infections: presumptive diagnosis based on the international microbiological criteria adopted in the state of São Paulo, Brazil, 2011-2014. J Bras Pneumol.

[12] Griffith DE, Aksamit T, Brown-Elliott BA, Catanzaro A, Daley C, Gordin F (2007). An official ATS/IDSA statement: diagnosis, treatment, and prevention of nontuberculous mycobacterial diseases. Am J Respir Crit Care Med.

[13] Loebinger MR, Quint JK, Laan R van der, Obradovic M, Chawla R, Kishore A (2023). Risk Factors for nontuberculous mycobacterial pulmonary disease: a systematic literature review and meta-analysis. Chest.

[14] Conyers LE, Saunders BM (2024). Treatment for non-tuberculous mycobacteria: challenges and prospects. Front Microbiol.

[15] Ministério da Saúde (2022). Manual e Recomendações para o Diagnóstico Laboratorial de Tuberculose e Micobactérias não Tuberculosas de Interesse em Saúde Pública no Brasil.

[16] Daley CL, Iaccarino JM, Lange C, Cambau E, Wallace RJ, Andrejak C (2020). Treatment of nontuberculous mycobacterial pulmonary disease: an official ATS/ERS/ESCMID/IDSA clinical practice guideline. Clin Infect Dis.

[17] Carneiro MS, Nunes LS, De David SMM, Dias CF, Barth AL, Unis G (2018). Nontuberculous mycobacterial lung disease in a high tuberculosis incidence setting in Brazil. J Bras Pneumol.

[18] Zamarioli LA, Coelho AGV, Pereira CM, Nascimento ACC, Ueki SYM, Chimara E (2008). Descriptive study of the frequency of nontuberculous mycobacteria in the Baixada Santista region of the state of São Paulo, Brazil. J Bras Pneumol.

[19] Duarte RS, Lourenço MCS, Fonseca LS, Leão SC, Amorim ELT, Rocha ILL (2009). Epidemic of postsurgical infections caused by *Mycobacterium massiliense*. J Clin Microbiol.

[20] CLSI (2018). Performance Standards for Antimicrobial Susceptibility Testing.

[21] Akalu TY, Clements ACA, Liyew AM, Gilmour B, Murray MB, Alene KA (2024). Risk factors associated with post-tuberculosis sequelae: a systematic review and meta-analysis. EClinicalMedicine.

[22] Hwang H, Lee JK, Heo EY, Kim DK, Lee HW (2023). The factors associated with mortality and progressive disease of nontuberculous mycobacterial lung disease: a systematic review and meta-analysis. Sci Rep.

[23] Dhasmana DJ, Whitaker P, van der Laan R, Frost F (2024). A practical guide to the diagnosis and management of suspected Non-tuberculous Mycobacterial Pulmonary Disease (NTM-PD) in the United Kingdom. Npj Prim Care Respir Med.

[24] Gupta KB, Gupta R, Atreja A, Verma M, Vishvkarma S (2009). Tuberculosis and nutrition. Lung India.

[25] Ratnatunga CN, Lutzky VP, Kupz A, Doolan DL, Reid DW, Field M (2020). The rise of non-tuberculosis mycobacterial lung disease. Front Immunol.

[26] Griffith DE, Brown-Elliott BA, Wallace RJ (2003). Thrice-weekly clarithromycin-containing regimen for treatment of *Mycobacterium kansasii* lung disease: results of a preliminary study. Clin Infect Dis.

[27] Winthrop KL, McNelley E, Kendall B, Marshall-Olson A, Morris C, Cassidy M (2010). Pulmonary nontuberculous mycobacterial disease prevalence and clinical features: an emerging public health disease. Am J Respir Crit Care Med.

[28] Haramati A, Eacobacci K, Turetz ML, Latson L, Brusca-Augello G, Girvin F (2025). Imaging features of nontuberculous mycobacterial pulmonary disease. Radiographics.

[29] Dos Anjos LRB, Parreira PL, Torres PPTS, Kipnis A, Junqueira-Kipnis AP, Rabahi MF (2020). Non-tuberculous mycobacterial lung disease: a brief review focusing on radiological findings. Rev Soc Bras Med Trop.

[30] Gopinath K, Singh S (2010). Non-tuberculous mycobacteria in TB-endemic countries: are we neglecting the danger?. PLoS Negl Trop Dis.

[31] Abbew ET, Lorent N, Mesic A, Wachinou AP, Obiri-Yeboah D, Decroo T (2024). Challenges and knowledge gaps in the management of non-tuberculous mycobacterial pulmonary disease in sub-Saharan African countries with a high tuberculosis burden: a scoping review. BMJ Open.

[32] Ueki SYM, Martins MC, Telles MAS, Virgilio MC, Giampaglia CMS, Chimara E (2005). Nontuberculous mycobacteria: species diversity in São Paulo state, Brazil. J Bras Patol Med Lab.

[33] Tarashi S, Sakhaee F, Masoumi M, Jajin MG, Siadat SD, Fateh A (2023). Molecular epidemiology of nontuberculous mycobacteria isolated from tuberculosis-suspected patients. AMB Express.

[34] Arttawejkul P, Kongpolprom N (2018). A case of pulmonary infection caused by *Mycobacterium asiaticum*: difficulties on diagnostic and therapeutic approaches. Respir Med Case Rep.

[35] Grech M, Carter R, Thomson R (2010). Clinical significance of *Mycobacterium asiaticum* isolates in Queensland, Australia. J Clin Microbiol.

[36] Lee JS, Lee JH, Yoon SH, Kim TS, Seong MW, Han SK (2017). Implication of species change of nontuberculous mycobacteria during or after treatment. BMC Pulm Med.

[37] Griffith DE, Aksamit TR (2012). Therapy of refractory nontuberculous mycobacterial lung disease. Curr Opin Infect Dis.

[38] Haworth CS, Floto RA (2017). Introducing the new BTS Guideline: Management of non-tuberculous mycobacterial pulmonary disease (NTM-PD). Thorax.

[39] Brown-Elliott BA, Bush G, Hughes MD, Rodriguez E, Weikel CA, Min SB (2024). *In vitro* activity of gepotidacin and comparator antimicrobials against isolates of nontuberculous mycobacteria (NTM). Antimicrob Agents Chemother.

[40] Griffith DE (2010). Nontuberculous mycobacterial lung disease. Curr Opin Infect Dis.

