# Supplementary material for: Pulmonary infection by non-tuberculous mycobacteria in an endemic region for tuberculosis in Northeast Brazil
Source: Braz J Med Biol Res. 2026 Jul 3;59:e15340. doi: 10.1590/1414-431X2026e15340 (PMC13331253; doi:10.1590/1414-431X2026e15340)
Supplement: Supplementary Material [file 1414-431X-bjmbr-59-e15340-suppl.pdf]

Table S1. Sociodemographic and clinical characteristics of pulmonary non-tuberculous mycobacteriosis (PNTM) and pulmonary tuberculosis (PTB) groups enrolled in the study.

Variables	PNTM n=21 (%)	PTB n=55 (%)	P
Sex			
Male	14 (67.7)	39 (70.9)	0.936
Female	7 (32.3)	16 (29.1)	
Age (years), mean ± SD	52.0 ± 14.7	41.0 ± 14.8	0.006*
Lifestyle			
Smoking	12 (57%)	13 (23.5)	0.012
Alcohol use	8 (38%)	10 (18.2)	0.127
Comorbidity			
Previous TB	14 (66.7)	6 (10.1)	<0.001
PLHIV	0 (0.0)	17 (30.9)	0.010
Cancer	1 (4.8)	2 (3.6)	–
COPD	1 (4.8)	0 (0.0)	–
Symptoms			
Cough			
Productive couch	14 (66.7)	34 (61.8)	–
Hemoptysis	1 (4.8%)	4 (7.3)	–
Weight loss	14 (66.7)	46 (83.6)	–
Fever	9 (42.8)	40 (72.7)	–

Data are reported as number (%), except for age. Chi-squared test; *t-test. (Shapiro-Wilk test: >0.05). SD: standard deviation; TB: tuberculosis; PLHIV: people living with human immunodeficiency virus; COPD: chronic obstructive pulmonary disease.

Table S2. The antimicrobial resistance profile of non-tuberculous mycobacteria isolates from biological samples of 15 patients with pulmonary non-tuberculous mycobacteria.

Antimicrobials	*M. abscessus subsp abscessus*			*M. fortuitum*			*M. abscessus subsp bolletii*			*M. kansasii*			*M. intracellulare*			Resistant strains per antimicrobial
	n=1	Profile	%	n=2	Profile	%	n=1	Profile	%	n=10	Profile	%	n=1	Profile	%	
Amikacin	0	S	0	0	S	0	0	S	0	0	S	0	0	S	0	0
Cefoxitin	1	R	100	0	S	0	0	S	0	–	–	–	–	–	–	1
Ciprofloxacin	1	R	100	0	S	0	1	R	100	3	R	30	0	S	0	5
Clarithromycin	1	R	100	1	R	50	1	R	100	1	R	10	0	S	0	4
Doxycycline	1	R	100	0	S	0	1	R	100	–	–	–	–	–	–	2
Moxifloxacin	0	S	0	0	S	0	1	R	100	0	S	0	0	S	0	1
Tobramycin	1	R	100	2	R	100	1	R	100	–	–	–	–	–	–	4
Sulfamethoxazole	1	R	100	2	R	100	1	R	100	4	R	40	1	R	100	9
Rifampicin	–			–			–			1	R	10	1	R	100	2
Ethambutol	–			–			–			5	R	50	1	R	100	6
Isoniazid	–			–			–			0	S	0	1	R	100	1
Linezolid	–			–			–			–	–	–	1	R	100	1
Streptomycin	–			–			–			4	R	40	1	R	100	5

R: resistant; S: susceptible.
